# Distal Adding-On as a Natural Shoulder Rebalancing Mechanism in Lenke Type 2A AIS with Right Sacral Slanting

**DOI:** 10.3390/jcm14196850

**Published:** 2025-09-27

**Authors:** Jae-Hyuk Yang, Jae Min Park, Hyukjune Seong, Chang Ju Hwang, Hyung Rae Lee

**Affiliations:** 1 Department of Orthopedic Surgery, Korea University Anam Hospital, Seoul 02841, Republic of Korea; kuspine@korea.ac.kr; 2 College of Medicine, Korea University, Seoul 02841, Republic of Korea; kikara02@korea.ac.kr (J.M.P.); sekil1@korea.ac.kr (H.S.); 3 Department of Orthopedic Surgery, Seoul Asan Medical Center, Seoul 05505, Republic of Korea; baski47@gmail.com

**Keywords:** adolescent idiopathic scoliosis (AIS), distal adding-on (DA), distal wedge angle (DWA), Lenke type 2A, Postoperative shoulder imbalance (PSI), radiologic shoulder height (RSH), sacral slanting

## Abstract

**Background/Objectives**: Distal adding-on (DA) is a common postoperative phenomenon in Lenke type 2A adolescent idiopathic scoliosis (AIS). Postoperative shoulder imbalance (PSI) is a clinically significant issue following AIS correction, as it may lead to aesthetic dissatisfaction, functional impairment, and reduced quality of life. This study investigated radiographic changes in DA and shoulder balance in Lenke type 2A AIS, particularly focusing on distal wedge angle (DWA) and radiologic shoulder height (RSH) in patients with right sacral slanting (RSS). **Methods**: We retrospectively analyzed 120 patients with Lenke type 2A AIS who underwent posterior spinal fusion. Patients were grouped by sacral slanting: right (RSS), left (LSS), or none (NS). Radiographic parameters including proximal thoracic curve angle, main thoracic curve angle, DWA, RSH were assessed at multiple time points. Univariate and multivariate linear regression analyses were used to identify factors associated with DA. **Results**: The RSS group consistently showed the highest DWA and the greatest incidence of DA. RSH initially exceeded the PSI threshold in all groups but decreased to approximately 10 mm by final follow-up. In the RSS group, the inverse relationship between increasing DWA and decreasing RSH was most pronounced. Univariate regression identified postoperative RSH and sacral slanting angle as significant predictors of DWA, though not in the final multivariate model. **Conclusions**: In Lenke type 2A AIS with RSS, an increasing DWA and decreasing RSH over time suggest that DA may serve as a compensatory mechanism for PSI. Sacral slanting and postoperative RSH may be relevant predictors of this dynamic alignment change.

## 1. Introduction

Distal adding-on (DA), a progressive extension of the curve below the level of instrumentation, is a common complication after surgical correction of adolescent idiopathic scoliosis (AIS) [1,2]. This can result in gradual loss of the initial correction, increased back pain, and reduced physical activity. Patients with distal adding-on may also experience lower satisfaction with surgical outcomes and, in some cases, require revision surgery [3,4]. Additionally, this complication can place extra stress on the lumbar spine, leading to further deformity or functional problems [5,6].

Distal adding-on seems to be particularly prevalent in patients with Lenke type 2A AIS, particularly in those exhibiting right sacral slanting (RSS). Lenke type 2A is defined by structural proximal thoracic curve (PTC) and main thoracic curve (MTC), with a non-structural lumbar curve [7]. Right sacral slanting refers to a rightward tilt of the upper surface of the sacrum, a feature that may alter spinal alignment and increase the risk of postoperative complications such as shoulder imbalance [8].

We assumed that distal adding-on may serve as a compensatory mechanism for natural shoulder rebalancing in patients with Lenke type 2A AIS and RSS. Several previous studies have suggested that distal adding-on may play a role in restoring postoperative shoulder balance in Lenke type 2A AIS with RSS. Cao et al. reported that patients who developed distal adding-on did not exhibit PSI, proposing that this phenomenon might contribute to improved shoulder balance [9]. Similarly, Hwang et al. demonstrated that patients with right sacral slanting experienced right-sided distal adding-on, which was accompanied by progressive improvement in PSI [10].

This study aims to investigate whether right-sided sacral slanting is associated with a higher incidence and greater magnitude of distal adding-on in patients with Lenke type 2 AIS. We also examine whether this increased occurrence of DA contributes to more significant correction of postoperative shoulder imbalance (PSI) over time. In addition, we analyze the risk factors for distal adding-on in patients with Lenke type 2A AIS. By clarifying these relationships, we hope to provide new insights into postoperative spinal dynamics in Lenke type 2 AIS.

## 2. Materials and Methods

### 2.1. Study Design and Patient Population

This retrospective study analyzed collected data from patients with Lenke type 2 AIS who underwent posterior spinal fusion at our institution between January 2010 and December 2020. Inclusion criteria were diagnosis of Lenke type 2 AIS, posterior spinal fusion with pedicle screw constructs, minimum 2-year follow-up, and availability of complete radiographic data. Patients with previous spine surgery, neuromuscular disorders, or congenital spinal anomalies were excluded. This retrospective cohort study was approved by the Institutional Review Board (IRB) of our institution (IRB number: 2024AN0597), and the requirement for informed consent was waived due to the retrospective nature of the study.

### 2.2. Demographic and Radiographic Measurements

Demographic data collected included age, sex, height, bone mineral density (BMD), weight, body mass index (BMI), and follow-up period. Skeletal maturity was assessed using the Risser grade (categorized as ≤2 or ≥3) [11].

Radiographic characteristics were gathered from patients’ clinical charts and the Picture Archiving and Communication System (PetaVision for Clinics, 3.1, Korea University Anam Hospital, Seoul, Republic of Korea). Radiological measurements included whole-spine standing anteroposterior radiographs taken preoperatively and postoperatively at immediate postoperative period, 1 month, 3 months, 6 months, 1 year, and 2 years after surgery, as well as at the final follow-up. The parameters measured were Cobb angles of the proximal and main thoracic curves, RSH, clavicle angle, T1 tilt, DWA, and sacral slanting angle.

**Figure 1:**
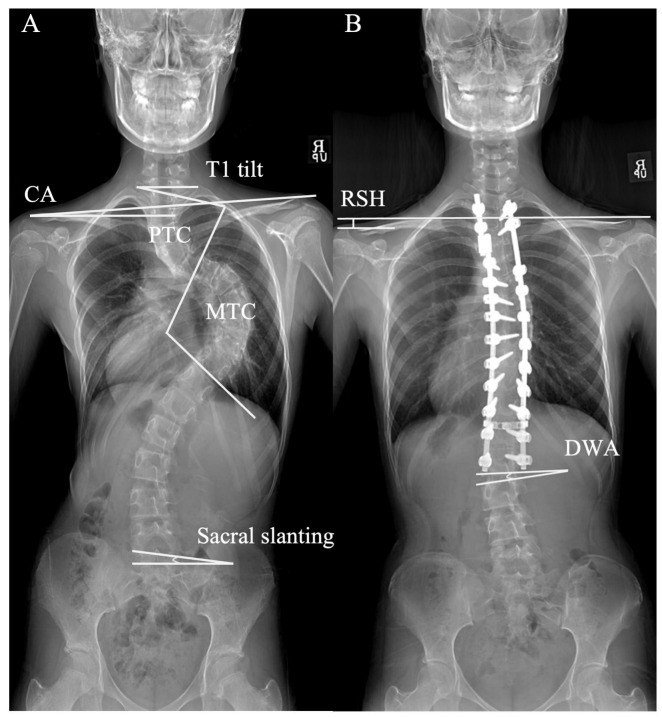
Measurements of radiologic parameters. (**A**) The T1 tilt, clavicle angle (CA), proximal thoracic curve (PTC) angle, main thoracic curve (MTC) angle, and sacral slanting angle are shown on standing whole-spine coronal pain radiograph. (**B**) Radiological shoulder height (RSH): the vertical distance difference between the acromial tips on both sides. Distal wedge angle (DWA): the angle formed between the superior endplate of the sacrum and a horizontal reference line. CA, clavicle angle; DWA, distal wedge angle; MTC, main thoracic curve; PTC, proximal thoracic curve; RSH, radiological shoulder height.

Clavicle angle was defined as the angulation of a horizontal line and the tangential line connecting the highest two points of each clavicle. T1 tilt angle was defined as the angulation of the upper endplate of T1 to the horizontal line. RSH was defined as the vertical difference between both acromion tips. Sacral slanting angle was defined as the angle formed between the superior endplate of the sacrum and a horizontal line; an angle over 3° was considered indicative of sacral slanting [12]. Positive values for RSH, clavicle angle, sacral slanting angle, and disc wedging angles indicated right-sided elevation. (Figure 1).

PSI was defined as an RSH ≥ 20 mm at the final follow-up [13]. DA was defined as either an increase of more than 5 mm in the deviation of the first vertebra below the lowest instrumented vertebra (LIV) from the central sacral vertical line (CSVL), or an increase of more than 5° in the angulation of the first disc below the LIV [14,15].

To ensure measurement reliability, all radiographic parameters were independently measured by two spine surgeons (H.R.L. and J.H.Y.), and the mean values were used for analysis. The interobserver reliability was excellent, with intraclass correlation coefficients (ICC) of 0.88 for DWA and 0.91 for RSH.

### 2.3. Surgical Technique

All surgeries were performed by a single senior surgeon using a standard posterior midline approach. Pedicle screws were inserted with a freehand technique under intraoperative C-arm fluoroscopic guidance to confirm accuracy. Fusion levels were selected based on established criteria, incorporating curve magnitude, structural rigidity, sagittal alignment, and shoulder balance. For the proximal fusion level, the size and flexibility of the PTC were the primary considerations. Specifically, when the PTC was relatively large and rigid on preoperative radiographs, fusion was extended proximally to T2. Conversely, when the PTC was smaller and more flexible, T3 or T4 was selected as the UIV. For the distal fusion level, the LIV was chosen based on CSVL alignment, disc wedging, and vertebral rotation, in accordance with established guidelines to minimize the risk of distal adding-on. Specifically, the LIV was selected as the stable vertebra (SV), defined as the vertebra most closely bisected by the CSVL on standing anteroposterior radiographs, or one level below (SV + 1) if significant disc wedging (>5°) or vertebral rotation (Nash-Moe grade ≥ 2) was observed below the SV [5,14,15]. Deformity correction was primarily achieved through the rod derotation technique, with supplemental segmental compression and distraction applied as required to optimize coronal and sagittal correction.

### 2.4. Statistical Analysis

Statistical analysis was performed using SPSS version 25.0 (IBM Corp., Armonk, NY, USA). Continuous variables were expressed as mean ± standard deviation, and categorical variables as frequencies and percentages. The chi-square test or Fisher’s exact test was used to compare categorical variables between groups. The independent *t*-test or Mann–Whitney U test was used for continuous variables, depending on the normality of data distribution. Pearson or Spearman correlation coefficients were calculated to assess the relationships between radiographic parameters. Multivariate logistic regression analysis was performed to identify independent risk factors for DA. A *p*-value < 0.05 was considered statistically significant.

## 3. Results

### 3.1. Baseline Demographics and Radiographic Characteristics

A total of 120 patients were included in the study and classified into three groups according to sacral slanting: 42 in the RSS group, 45 in the NS group, and 33 in the LSS group (Table 1).

There were no statistically significant differences among the three groups in baseline demographic variables, including age (RSS, NS, LSS: 15.3 ± 3.2, 15.1 ± 3.5, 15.2 ± 3.1 years, *p* = 0.93), sex ratio (35:7, 32:13, 24:9; *p* = 0.28), height (161.0 ± 7.6, 160.2 ± 7.9, 159.3 ± 8.0 cm; *p* = 0.68), weight (49.9 ± 9.7, 48.7 ± 8.6, 47.8 ± 8.5 kg; *p* = 0.53), body mass index (19.8 ± 0.4, 20.4 ± 0.2, 19.8 ± 0.3 kg/m^2^; *p* = 0.14), and bone mineral density T-score (0.5 ± 0.1, 0.4 ± 0.8, 0.3 ± 0.1; *p* = 0.33). The mean follow-up duration was also similar among the groups (52.8 ± 25.1, 54.2 ± 23.4, 49.7 ± 26.8 months; *p* = 0.66).

Radiographic characteristics likewise demonstrated no statistically significant intergroup differences. Proximal thoracic curve (PTC) was 40.6 ± 11.6, 39.1 ± 9.8, and 42.3 ± 10.1° (*p* = 0.59) in RSS, NS, and LSS groups, respectively; main thoracic curve (MTC), 63.8 ± 11.2, 62.5 ± 10.9, and 61.7 ± 12.4° (*p* = 0.76); and PTC/MTC ratio, 68.8 ± 10.9, 64.6 ± 11.5, and 66.1 ± 12.2 (*p* = 0.32). There were no significant differences in RSH (−8.1 ± 7.5, −9.2 ± 9.0, −10.3 ± 8.7 mm; *p* = 0.49), clavicle angle (−1.4 ± 1.8, −1.6 ± 2.1, −1.5 ± 2.0°; *p* = 0.85), or T1 tilt (3.9 ± 5.1, 3.7 ± 4.8, 4.3 ± 5.5°; *p* = 0.80) among the three groups. The distribution of Risser grade (≤2 : ≥3; 12:30, 15:30, 6:27; *p* = 0.69) and sacral slanting angle (1.0 ± 1.4, 0.4 ± 2.1, 0.2 ± 2.4°; *p* = 0.47) were also comparable between groups.

### 3.2. Distal Adding-On and Postoperative Shoulder Balance over Time

Table 2 showed significant differences in both DWA and RSH among the RSS, NS, and LSS groups at all measured time points.

**Figure 2:**
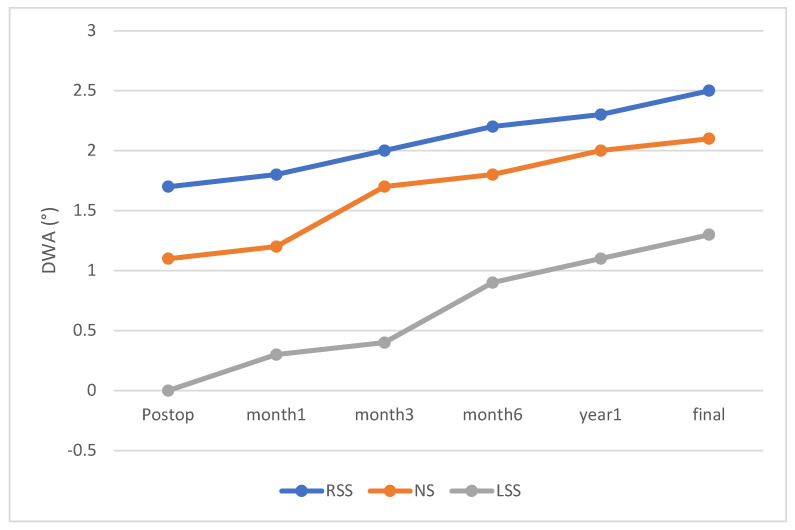
Time-dependent Changes in Distal Wedge Angle. Time-dependent changes in the distal wedge angle are compared among the RSS, NS, and LSS groups. Statistically significant differences among the RSS, NS, and LSS groups were observed at all time points (*p* < 0.05).

At immediate postoperative assessment, the mean DWA values were −0.1 ± 0.5° in the LSS group, 1.1 ± 0.4° in the NS group, and 1.7 ± 0.6° in the RSS group. DWA progressively increased over the follow-up period in all groups, with the RSS group consistently demonstrating the highest angles, followed by the NS group and then the LSS group at each visit (final follow-up: RSS, 2.5 ± 0.9°; NS, 2.1 ± 0.8°; LSS, 1.3 ± 0.7°; *p* < 0.01) (Figure 2). DA occurred in 47.6% of patients in the RSS group, which was higher than in the NS (28.9%) and LSS (18.2%) groups (*p* = 0.02).

RSH also exhibited significant intergroup differences at all postoperative intervals. Immediately after surgery, mean RSH was 22.4 ± 2.5 mm in the RSS group, 21.2 ± 3.1 mm in the NS group, and 20.6 ± 2.8 mm in the LSS group. Although RSH decreased over time in every group, the RSS group maintained the highest values at each time point, while the LSS group showed the lowest. At the final follow-up, the mean RSH was 8.0 ± 2.6 mm in the RSS group, 9.4 ± 2.9 mm in the NS group, and 10.2 ± 3.0 mm in the LSS group (*p* < 0.01).

**Figure 3:**
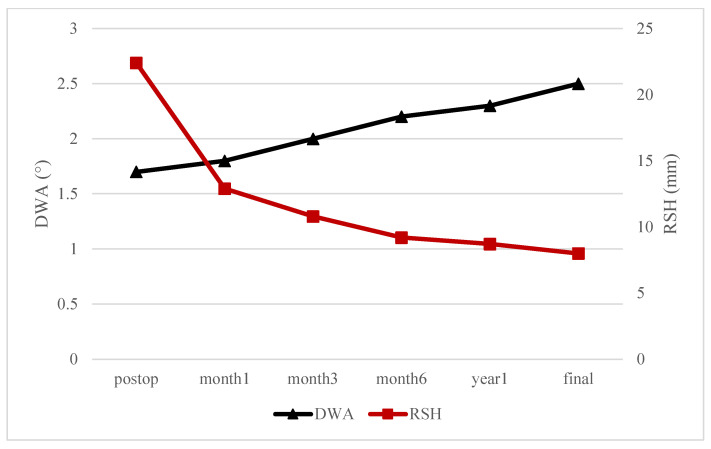
Time-dependent Changes in Distal Wedge Angle (DWA) and Radiological Shoulder Height (RSH) in RSS group.

In the RSS group, Figure 3 illustrates a divergent pattern in the postoperative changes in DWA and RSH over time. Specifically, DWA demonstrated a continuous increase throughout the follow-up period, whereas RSH exhibited a steady decrease.

To evaluate the evolution of DA as a compensatory mechanism for PSI, we assessed the stability of DA within the follow-up period (mean 2.1 ± 0.4 years). Among the 31 patients with DA (47.6% in RSS, 28.9% in NS, 18.2% in LSS; *p* = 0.02), 22 (70.9%) exhibited stable DA, defined as a DWA change of ≤0.5° between the 1-year and 2-year follow-up radiographs. Nine patients (29.1%) showed progressive DA, defined as a DWA increase larger than 0.5° during this period. In the RSS group, 14 of 20 DA cases (70.0%) were stable, while 6 (30.0%) were progressive. In the NS group, 7 of 9 DA cases (77.8%) were stable, and in the LSS group, all 4 DA cases (100%) were stable. Importantly, no patients required revision surgery for DA or PSI within the 2-year follow-up period.

### 3.3. Regression Analyses

Univariate and multivariate linear regression analyses were performed to identify risk factors associated with distal adding-on in Lenke type 2A AIS (Table 3). On univariate analysis, postoperative radiologic shoulder height (post RSH; β = 0.04, *p* = 0.03) and sacral slanting (β = 0.29, *p* = 0.04) were found to be significantly associated with distal adding-on in Lenke type 2A AIS. No other demographic or radiographic variables, including age, preoperative curve magnitudes, and postoperative CSVL, reached statistical significance. However, in the final multivariate model, there were no statistically significant predictors of distal adding-on identified in the multivariate analysis (post RSH, *p* = 0.05; sacral slanting, *p* = 0.06).

## 4. Discussion

In this study, we showed two key findings regarding postoperative spinal dynamics in patients with Lenke type 2A AIS, especially those with RSS. First, our results confirmed that RSS is associated with both a higher incidence and greater magnitude of DA following posterior spinal fusion. Second, we demonstrated that DA may function as a compensatory mechanism facilitating gradual shoulder balance correction over time.

In Table 2, DWA showed a consistent progressive increase in all three groups throughout the follow-up period, with the RSS group showing the highest values at each time point. The final follow-up data demonstrated significantly higher DWA values in the RSS group (2.5 ± 0.9°) compared to the NS and LSS groups (2.1 ± 0.8° and 1.3 ± 0.7°, respectively; *p* < 0.01). Moreover, the incidence of DA was also notably higher in the RSS group (47.6%) than in the NS (28.9%) and LSS (18.2%) groups (*p* = 0.02). These findings indicate that DA is particularly common in Lenke type 2A AIS patients with RSS, underscoring the potential biomechanical influence of sacral morphology on postoperative spinal behavior.

In contrast to these DWA trends, RSH showed a marked and consistent decline over time after surgery. Immediately after surgery, all groups exhibited high RSH values, exceeding the PSI threshold (≥20 mm) (RSS, 22.4 ± 2.5; NS, 21.2 ± 3.1; LSS, 20.6 ± 2.8 mm; *p* = 0.03). However, over time, RSH values gradually decreased in all groups, eventually reaching approximately 10 mm at the final follow-up (RSS, 8.0 ± 2.6; NS, 9.4 ± 2.9; LSS, 10.2 ± 3.0 mm; *p* < 0.01) (Table 2). This gradual postoperative decline in RSH appears to reflect a natural shoulder rebalancing [16,17]. Li et al. reported a similar phenomenon, noting that while 13 patients (52%) exhibited shoulder imbalance before surgery, only 4 patients (16%) showed mild shoulder imbalance postoperatively, suggesting a tendency toward spontaneous correction of shoulder asymmetry over time [18].

**Figure 4:**
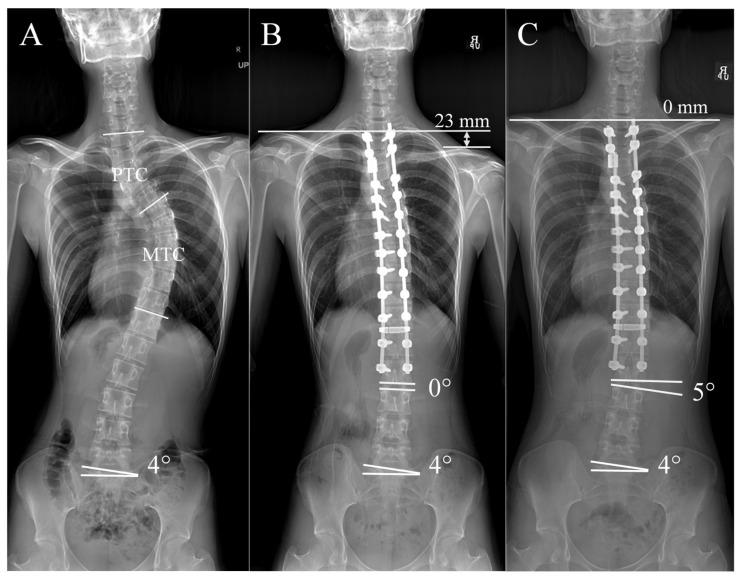
Representative case of a patient with Lenke 2A AIS from the RSS group. (**A**) The patient showed a right-sided sacral slanting of 4° preoperatively. (**B**) Immediate postoperative radiograph after surgical correction, demonstrating improved spinal alignment, a radiological shoulder height (RSH) difference of 23 mm, and a neutral distal wedge angle (DWA) of 0°. (**C**) At 2 years follow-up, showing natural shoulder rebalancing and a DWA of 5°, indicating distal adding-on.

The RSS group showed a more marked pattern, with a continuous increase in DWA accompanied by a steady reduction in RSH (Figure 3). This relationship is visualized in Figure 4. The patient had 4° of right sacral slanting. The immediate postoperative radiograph showed an RSH of 23 mm, indicative of significant right shoulder depression and PSI, while the DWA was neutral at 0°. At two-year follow-up, the RSH had normalized to 0 mm and the DWA had increased to 5°, suggesting that the right shoulder had elevated while the distal spine underwent additional wedging. This finding supports the interpretation that distal adding-on may act as a compensatory mechanism facilitating natural shoulder rebalancing over time [19].

**Figure 5:**
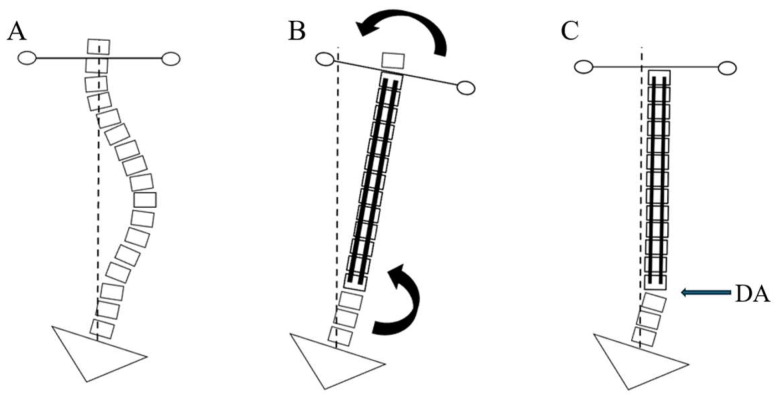
Illustration of how right sacral slanting (RSS) contributes to distal adding-on (DA) over time. (**A**) Preoperative spinal alignment with right sacral slanting, indicating a biomechanical predisposition for further wedging at the lower vertebral segments. (**B**) Following immediate correction, the proximal thoracic curve (PTC) is straightened, leading to right-sided shoulder depression. Subsequently, as the shoulders tend toward natural rebalancing, a rightward force is exerted at the distal wedging immediately below the instrumented segments (arrow). (**C**) Final postoperative alignment demonstrating distal adding-on (DA, arrow) development under the right-sided sacral slanting influence, potentially serving as a compensatory mechanism to maintain or improve shoulder balance.

These results suggest that the development of DA, reflected by the increasing DWA, may represent a process of natural shoulder rebalancing rather than solely a postoperative complication. As illustrated in Figure 5, immediate postoperative correction of the PTC often results in right shoulder depression, particularly in patients with RSS [20,21]. Over time, a corrective force aiming to elevate the right shoulder acts concurrently with a downward force induced by sacral slanting, leading to right-sided distal wedging below the instrumented segments. This biomechanical adaptation could facilitate gradual shoulder leveling and improve coronal alignment without additional surgical intervention.

In contrast to the RSS group, the LSS group exhibited a less pronounced increase in DWA (from −0.1 ± 0.5° to 1.3 ± 0.7°) and a smaller reduction in RSH (from 20.6 ± 2.8 mm to 10.2 ± 3.0 mm) during follow-up (Table 2). These findings suggest that the compensatory relationship between DA and RSH was less evident in the LSS group. This trend contrasts with the RSS group, in which Figure 3 demonstrates a clear inverse association between increasing DWA and decreasing RSH, supporting the hypothesis that right-sided DA may function as a rebalancing mechanism for PSI [9,10]. Several factors may help explain the weaker relationship observed in LSS. First, the mean sacral slanting angle was lower in the LSS group than in the RSS group (0.2 ± 2.4° vs. 1.0 ± 1.4°, Table 1), suggesting that the biomechanical impact of the leftward tilt may have been insufficient to drive significant distal compensatory changes [8]. Second, the LSS group demonstrated consistently lower DWA values across all postoperative time points (Table 2), indicating that the degree of distal wedging was limited. Finally, it is possible that leftward sacral slanting creates a distal spinal angle that biomechanically opposes the corrective direction typically required to elevate the depressed right shoulder in Lenke type 2A AIS [20,21]. This configuration may inherently inhibit the development of DA and reduce its effectiveness in restoring shoulder balance. Taken together, these findings imply that not only the magnitude but also the direction of sacral slanting significantly influence postoperative spinal behavior, and further biomechanical studies are needed to better understand these asymmetrical responses.

Based on these observations, we analyzed potential risk factors for DA in Lenke type 2A AIS patients. As shown in Table 3, univariate linear regression analysis identified postoperative RSH and sacral slanting as significant risk factors associated with DA occurrence, suggesting a possible link between postoperative shoulder balance and distal curve behavior. Although these factors did not reach statistical significance in the final multivariate model, previous studies have also reported associations between RSH, sacral slanting, and DA, supporting their potential relevance in postoperative alignment dynamics [9].

Our mid-term follow-up (mean 2.1 ± 0.4 years) suggests that DA may act as a benign compensatory mechanism for PSI, particularly in the RSS group, with no patients requiring revision surgery within this period. However, the long-term evolution of DA remains uncertain, as some cases showed progressive DA, which could lead to clinically significant deformity or require surgical intervention [15]. Future studies with extended follow-up are needed to assess DA’s stability, progression, and impact on outcomes like pain or function.

PSI remains one of the most significant concerns following corrective surgery for AIS, as it has been closely associated with adverse cosmetic and functional outcomes [22]. In particular Lenke type 2A AIS, postoperative left shoulder elevation, often resulting from insufficient proximal thoracic curve correction or underlying sacral asymmetry, can significantly diminish patient satisfaction despite adequate spinal alignment [23,24,25]. These aesthetic and functional consequences emphasize the importance of meticulous surgical planning focused on achieving balanced shoulder alignment and highlight the need for long-term follow-up strategies to manage PSI effectively.

Given the potential for PSI to cause adverse clinical outcomes, it can be argued that if PSI is naturally rebalanced through increased DWA, as seen in DA, then DA should not necessarily be regarded as purely negative. In line with our interpretation, recent studies have also begun to propose that DA may contribute to postoperative shoulder balance maintenance in certain subgroups, including Lenke type 2A patients with sacral slanting [9,26]. For example, Hwang et al. showed that DA could contribute to improved postoperative shoulder balance in select AIS patients, supporting this evolving perspective. This evolving perspective highlights the need for individualized interpretation of DA within the broader context of postoperative spinal and shoulder alignment.

In addition to sacral slanting, the selection of the upper instrumented vertebra (UIV) is well known to strongly influence postoperative shoulder balance. In our cohort, the UIV was determined primarily by the size and flexibility of the PTC, extending to T2 in cases with a large and rigid curve and to T3/T4 when the PTC was relatively small and flexible. This principle is in line with classical studies emphasizing the role of proximal thoracic curve fusion in achieving balanced shoulders. More recently, alternative methods such as the modified S-line [27] and validation studies on UIV recommendations [28] have provided additional guidance for optimal level selection.

Despite the strengths of this study, several limitations should be acknowledged. First, the sample size of each group was relatively modest, which may limit the statistical power and generalizability of our findings. Second, due to the retrospective nature of the study, the possibility of selection bias cannot be excluded, and causal inferences between sacral slanting, RSH, and distal adding-on (DA) should be interpreted with caution. Third, although the follow-up period was sufficient to evaluate mid-term postoperative changes, it may not adequately reflect the long-term stability of shoulder balance or the progression of coronal deformity. While our analysis revealed that most cases of DA remained stable within the 2-year follow-up period and no patients required revision surgery, a subset of patients exhibited progressive DA, the long-term clinical significance of which remains unknown. The compensatory role of DA for PSI appears promising; however, further investigations with extended follow-up are warranted to determine whether persistent or progressive DA may contribute to spinal imbalance, axial symptoms, or eventual surgical indication. Fourth, the selection of the UIV and LIV was not standardized but based on established surgical principles, including PTC size and flexibility for UIV (T2 for large, rigid curves; T3/T4 for smaller, flexible curves) and CSVL alignment, disc wedging (>5°), and vertebral rotation (Nash-Moe grade ≥ 2) for LIV [5,14,15,27,28]. While these criteria reflect clinical practice, variability in UIV and LIV selection may have influenced postoperative shoulder balance (RSH) and DA. Previous studies have noted that UIV and LIV choices can significantly impact shoulder balance and the risk of DA in Lenke type 2A AIS [11,28]. However, this study focused primarily on the role of sacral slanting in postoperative spinal dynamics, and thus, a detailed evaluation of UIV and LIV effects was beyond the scope of this analysis. This focus on sacral slanting may limit our ability to fully elucidate the contributions of UIV and LIV selection to RSH and DA.

Moreover, this study relied exclusively on radiographic parameters, such as RSH and DWA, to assess PSI and DA. While these objective measures are valuable, they do not fully capture the patient’s perception of aesthetic or functional outcomes. Additionally, measurements such as DWA and sacral slanting may be influenced by unaccounted confounding factors, including pelvic rotation, leg length discrepancy, and patient positioning. Future prospective studies incorporating larger sample sizes, longer follow-up, and comprehensive evaluation tools—including both objective radiographic and subjective clinical assessments—are essential to validate and expand upon the current findings.

These findings offer a more integrative perspective on postoperative compensation mechanisms in Lenke type 2A AIS. While previous studies have separately reported associations between shoulder imbalance and distal adding-on, or between sacral slanting and shoulder balance, our results suggest a sequential relationship in which right sacral slanting promotes distal adding-on, which in turn contributes to spontaneous right shoulder elevation. This pattern, most evident in RSS patients with synchronous changes in DWA and RSH (Figure 5), may help explain how certain patients achieve postoperative shoulder balance without extended proximal fusion. Recognizing this dynamic may assist surgeons in anticipating alignment changes and making more tailored decisions regarding fusion levels.

## 5. Conclusions

In Lenke type 2A AIS, patients with right sacral slanting showed a consistent pattern of increasing DWA and decreasing RSH over time. These findings suggest that distal adding-on may play a compensatory role in restoring shoulder balance, rather than representing a solely negative postoperative phenomenon. Sacral slanting and postoperative RSH were identified as potential risk factors for distal adding-on, underscoring the need for careful surgical planning and long-term monitoring in patients with sacral asymmetry.

## Figures and Tables

**Table 1:** Baseline Demographics and Radiographic Characteristics by Sacral Slanting Group.

	RSS (*N* = 42)	NS (*N* = 45)	LSS (*N* = 33)	*p*
Age (year)	15.3 ± 3.2	15.1 ± 3.5	15.2 ± 3.1	0.93
Sex (M:F)	35:7	32:13	24:9	0.28
Height (cm)	161.0 ± 7.6	160.2 ± 7.9	159.3 ± 8.0	0.68
Weight (kg)	49.9 ± 9.7	48.7 ± 8.6	47.8 ± 8.5	0.53
BMI (kg/m^2^)	19.8 ± 0.4	20.4 ± 0.2	19.8 ± 0.3	0.14
BMD, T-score	0.5 ± 0.1	0.4 ± 0.8	0.3 ± 0.1	0.33
Follow-up (month)	52.8 ± 25.1	54.2 ± 23.4	49.7 ± 26.8	0.66
PTC (°)	40.6 ± 11.6	39.1 ± 9.8	42.3 ± 10.1	0.59
MTC (°)	63.8 ± 11.2	62.5 ± 10.9	61.7 ± 12.4	0.76
PTC/MTC ratio	68.8 ± 10.9	64.6 ± 11.5	66.1 ± 12.2	0.32
RSH (mm)	−8.1 ± 7.5	−9.2 ± 9.0	−10.3 ± 8.7	0.49
Clavicle angle (°)	−1.4 ± 1.8	−1.6 ± 2.1	−1.5 ± 2.0	0.85
T1 tilt (°)	3.9 ± 5.1	3.7 ± 4.8	4.3 ± 5.5	0.80
Risser grade, ≤2 : ≥3	12:30	15:30	6:27	0.69
Sacral slanting (°)	1.0 ± 1.4	0.4 ± 2.1	0.2 ± 2.4	0.47

BMD, bone mineral density; BMI, body mass index; PTC, proximal thoracic curve; MTC, main thoracic curve; RSH, radiologic shoulder height.

**Table 2:** Comparison of Postoperative Shoulder Imbalance, Distal wedge angle and Distal adding-on occurrence.

	RSS (*N* = 42)	NS (*N* = 45)	LSS (*N* = 33)	*p*
DWA (°)				
Postop immediate	1.7 ± 0.6	1.1 ± 0.4	−0.1 ± 0.5	<0.01 *
Postop 1 M	1.8 ± 0.7	1.2 ± 0.5	0.3 ± 0.5	0.01 *
Postop 3 M	2.0 ± 0.7	1.7 ± 0.6	0.4 ± 0.6	<0.01 *
Postop 6 M	2.2 ± 0.8	1.8 ± 0.7	0.9 ± 0.6	<0.01 *
Postop 1 Y	2.3 ± 0.9	2.0 ± 0.7	1.1 ± 0.6	<0.01 *
Postop 2 Y	2.5 ± 0.9	2.1 ± 0.8	1.3 ± 0.7	<0.01 *
DA Occurrence	20 (47.6%)	13 (28.9%)	6 (18.2%)	0.02 *
RSH (mm)				
Postop immediate	22.4 ± 2.5	21.2 ± 3.1	20.6 ± 2.8	0.03 *
Postop 1 M	12.9 ± 2.0	14.6 ± 2.3	15.1 ± 2.4	0.04 *
Postop 3 M	10.8 ± 1.8	13.2 ± 2.1	14.3 ± 2.2	0.02 *
Postop 6 M	9.2 ± 2.3	11.7 ± 2.5	12.9 ± 2.6	<0.01 *
Postop 1 Y	8.7 ± 2.4	10.5 ± 2.6	11.4 ± 2.8	<0.01 *
Postop 2 Y	8.0 ± 2.6	9.4 ± 2.9	10.2 ± 3.0	<0.01 *

RSH, radiologic shoulder height; DA, distal adding; DWA, distal wedge angle. * *p* < 0.05, statistically significant differences.

**Table 3:** Univariate and multivariate linear regression analysis for risk factors associated with distal adding-on in Lenke type 2A AIS.

Variables	Beta	SE	t-Value	95% CI	*p*-Value
Univariate Analysis					
Age	−0.01	0.01	−1.33	−0.02–0.00	0.19
Pre MTC	0	0.02	0.20	−0.04–0.05	0.84
Flexibility passive	0	0.02	0.25	−0.03–0.04	0.80
Pre PTC	−0.02	0.03	−0.54	−0.08–0.04	0.59
Pre T1tilt	−0.06	0.05	−1.20	−0.16–0.04	0.23
Pre CSVL	0	0.02	−0.13	−0.05–0.04	0.90
Pre RSH	0.03	0.03	1.31	−0.02–0.08	0.19
Post CSVL	0.01	0.02	0.45	−0.03–0.05	0.66
Post RSH	0.04	0.02	2.18	0.00–0.07	0.03 *
Sacral slanting	0.29	0.14	2.10	0.02–0.57	0.04 *
Finally Selected Model					
(Intercept)	0.26	0.42	0.61	−0.57–1.09	0.54
Post RSH	0.03	0.02	1.97	−0.00–0.07	0.05
Sacral slanting	0.26	0.14	1.87	−0.02–0.54	0.06

PTC, proximal thoracic curve; MTC, main thoracic curve; CSVL, central sacral vertical line; RSH, radiologic shoulder height. * *p* < 0.05, statistically significant differences.

## Data Availability

The datasets used and/or analysed during the current study are available from the corresponding author upon reasonable request.

## Abbreviations

The following abbreviations are used in this manuscript:

AIS	Adolescent Idiopathic Scoliosis
DA	Distal Adding-on
DWA	Distal Wedge Angle
PSI	Postoperative Shoulder Imbalance
RSS	Right Sacral Slanting
RSH	Radiologic Shoulder Height
LSS	Left Sacral Slanting
NS	None (No Sacral Slanting)
PTC	Proximal Thoracic Curve
MTC	Main Thoracic Curve
UIV	Upper Instrumented Vertebra
LIV	Lowest Instrumented Vertebra
CSVL	Central Sacral Vertical Line
ICC	Intraclass Correlation Coefficients
IRB	Institutional Review Board
BMD	Bone Mineral Density
BMI	Body Mass Index

## References

[1] Roye B.D., Matsumoto H., Fano A.N., Marciano G.F., Iyer R.R., Boby A., Bainton N., Lenke L.G., Newton P.O., Vitale M.G. (2022). Distal adding-on in adolescent idiopathic scoliosis results in diminished health-related quality of life at 10 years following posterior spinal fusion. Spine Deform..

[2] Suk S.-I., Lee S.-M., Chung E.-R., Kim J.-H., Kim W.-J., Sohn H.-M. (2003). Determination of Distal Fusion Level With Segmental Pedicle Screw Fixation in Single Thoracic Idiopathic Scoliosis. Spine.

[3] Li Y., Li J., Luk K.D.K., Zhang C., Sun J., Wang G. (2023). Relationship between Fusion Mass Shift and Postoperative Distal Adding-on in Lenke 1 Adolescent Idiopathic Scoliosis after Selective Thoracic Fusion. Asian Spine J..

[4] Kim H., Chang B.S., Chang S.Y. (2024). Current issues in the treatment of adolescent idiopathic scoliosis: A comprehensive narrative review. Asian Spine J..

[5] Cao K., Watanabe K., Kawakami N., Tsuji T., Hosogane N., Yonezawa I., Machida M., Yagi M., Kaneko S., Toyama Y. (2014). Selection of Lower Instrumented Vertebra in Treating Lenke Type 2A Adolescent Idiopathic Scoliosis. Spine.

[6] Wang Y., Bünger C.E., Zhang Y., Wu C., Li H., Hansen E.S. (2014). Distal Adding on in Lenke 1A Scoliosis: What Causes It? How Can It Be Prevented?. Spine Deform..

[7] Lenke L.G. (2007). The Lenke Classification System of Operative Adolescent Idiopathic Scoliosis. Neurosurg. Clin. N. Am..

[8] Cho J.H., Lee C.S., Joo Y.S., Park J., Hwang C.J., Lee D.H. (2017). Association between Sacral Slanting and Adjacent Structures in Patients with Adolescent Idiopathic Scoliosis. Clin. Orthop. Surg..

[9] Cao K., Watanabe K., Hosogane N., Toyama Y., Yonezawa I., Machida M., Yagi M., Kaneko S., Kawakami N., Tsuji T. (2014). Association of Postoperative Shoulder Balance With Adding-on in Lenke Type II Adolescent Idiopathic Scoliosis. Spine.

[10] Hwang C.J., Lee H.R., Lee S.K., Seok S.Y., Cho J.H., Lee D.-H., Lee C.S. (2024). Does Sacral Slanting Affect Postoperative Shoulder Balance in Patients With Lenke Type 2A Adolescent Idiopathic Scoliosis?. Neurospine.

[11] Isogai N., Yagi M., Otomo N., Maeda Y., Suzuki S., Nori S., Tsuji O., Nagoshi N., Okada E., Fujita N. (2023). Upper End Vertebra of Proximal Thoracic Curve At T1 is a Novel Risk Factor of Postoperative Shoulder Imbalance in Lenke Type 2 Adolescent Idiopathic Scoliosis. Glob. Spine J..

[12] Yamauchi I., Nakashima H., Ito S., Segi N., Ouchida J., Tauchi R., Ohara T., Kawakami N., Imagama S. (2023). Wedge-Shaped Deformity of the First Sacral Vertebra Associated with Adolescent Idiopathic Scoliosis: A Comparison of Cases with and without Scoliosis. Spine Surg. Relat. Res..

[13] Hadgaonkar S., Shah S., Bhilare P., Kothari A., Shyam A., Sancheti P., Aiyer S.N. (2020). Clinical and radiological factors associated with postoperative shoulder imbalance and correlation with patient-reported outcomes following scoliosis surgery. J. Orthop..

[14] Shu S., Bao H., Zhang Y., Gu Q., Zhang T., Jing W., Liu Z., Qiu Y., Zhu Z. (2020). Selection of Distal Fusion Level for Lenke 5 Curve: Does the Rotation of the Presumed Lower Instrumented Vertebra Matter?. Spine.

[15] Cho R.H., Yaszay B., Bartley C.E., Bastrom T.P., Newton P.O. (2012). Which Lenke 1A Curves Are at the Greatest Risk for Adding-On... and Why?. Spine.

[16] Moorthy V., Goh G.S., Guo C.M., Tan S.B., Chen J.L., Soh R.C.C. (2022). Shoulder Balance Following Correction Surgery for Adolescent Idiopathic Scoliosis: When Is It Achieved and Does the Type of Construct Matter?. Clin. Spine Surg..

[17] Okubo T., Konomi T., Yanai Y., Kobayashi Y., Furukawa M., Fujiyoshi K., Asazuma T., Yato Y. (2023). Incidence and predictive factors of shoulder imbalance after selective anterior spinal fusion surgery in Lenke type 5C adolescent idiopathic scoliosis. N. Am. Spine Soc. J..

[18] Li M., Gu S., Ni J., Fang X., Zhu X., Zhang Z. (2009). Shoulder balance after surgery in patients with Lenke Type 2 scoliosis corrected with the segmental pedicle screw technique. J. Neurosurg. Spine.

[19] Qin X., Xia C., Xu L., Sheng F., Yan H., Qiu Y., Zhu Z. (2018). Natural History of Postoperative Adding-On in Adolescent Idiopathic Scoliosis: What Are the Risk Factors for Progressive Adding-On?. BioMed Res. Int..

[20] Nikouei F., Ghandhari H., Ameri E., Mokarami F. (2022). Shoulder Imbalance in Adolescent Idiopathic Scoliosis: A Systematic Review of the Current State of the Art. Arch. Bone Jt. Surg..

[21] Lee H.R., Hwang C.J., Seok S.Y., Kim G.J., Cho J.H., Lee D.-H., Lee C.S. (2024). Shoulder balance in Lenke type 2 adolescent idiopathic scoliosis: Correlations among radiological indices, cosmetic indices, and patient-reported outcomes. J. Neurosurg. Spine.

[22] Qiu X.S., Ma W.W., Li W.G., Wang B., Yu Y., Zhu Z.Z., Qian B.P., Zhu F., Sun X., Ng B.K. (2009). Discrepancy between radiographic shoulder balance and cosmetic shoulder balance in adolescent idiopathic scoliosis patients with double thoracic curve. Eur. Spine J..

[23] Yang Y., Yang M., Zhao J., Zhao Y., Yang C., Li M. (2019). Postoperative shoulder imbalance in adolescent idiopathic scoliosis: Risk factors and predictive index. Eur. Spine J..

[24] Lee C.S., Hwang C.J., Lee D.H., Cho J.H. (2019). Does fusion to T2 compared with T3/T4 lead to improved shoulder balance in adolescent idiopathic scoliosis with a double thoracic curve?. J. Pediatr. Orthop. B.

[25] Eun I.S., Goh T.S., Kim D.S., Choi M., Lee J.S. (2023). Comparison of Korean Body Image Questionnaires in Adolescent Idiopathic Scoliosis. Asian Spine J..

[26] Kinoshita Y., Matsumura A., Namikawa T., Hoshino M., Hori Y., Nakamura H. (2024). Analyzing Factors Associated with Postoperative Shoulder Imbalance in Lenke2 Adolescent Idiopathic Scoliosis (Retrospective Cohort Study). World Neurosurg..

[27] Mimura T., Ikegami S., Banno T., Seki S., Ohba T., Oba H., Kuraishi S., Uehara M., Munakata R., Takizawa T. (2022). Usefulness of modified S-line for upper instrumented vertebra selection in adolescent idiopathic scoliosis Lenke type 2 curves. Sci. Rep..

[28] Bjerke B.T., Cheung Z.B., Shifflett G.D., Iyer S., Derman P.B., Cunningham M.E. (2015). Do Current Recommendations for Upper Instrumented Vertebra Predict Shoulder Imbalance? An Attempted Validation of Level Selection for Adolescent Idiopathic Scoliosis. HSS J..

